# Multifaceted mechanisms by which environmental endocrine-disrupting chemicals promote cancer progression: crosstalk among carcinogenesis, immunity, and metabolic reprogramming: narrative mini-review

**DOI:** 10.3389/fmolb.2026.1839082

**Published:** 2026-06-03

**Authors:** Aoran Du, Qiwei Yang, Lin Yu, Yishan Peng, Tao Huang, Qing Yuan, Wei Wang, Genshu Wang

**Affiliations:** 1 Department of Pulmonary and Critical Care Medicine, The Second Affiliated Hospital, School of Medicine, The Chinese University of Hong Kong, Shenzhen and Longgang District People’s Hospital of Shenzhen, Shenzhen, China; 2 The Second Clinical College of Guangzhou University of Chinese Medicine, Guangzhou, China; 3 State Key Laboratory of Traditional Chinese Medicine Syndrome, Department of Liver Transplant and Surgery of Guangdong Provincial Hospital of Chinese Medicine, Guangdong Provincial Engineering Research Center of Precicion Intelligent Surgical Equipment, The Second Clinical Medical College of Guangzhou University of Chinese Medicine, Guangzhou, China

**Keywords:** carcinogenesis, endocrine-disrupting chemicals, Immunity, metabolic reprogramming, mixture exposure

## Abstract

Endocrine-disrupting chemicals (EDCs) are ubiquitous and highly persistent environmental contaminants. Human exposure occurs through multiple routes, including dietary ingestion, water consumption, inhalation, and dermal contact, posing sustained health risks characterized by chronic, low-dose, and cumulative mixture exposure. Through interference with endocrine signaling and related biological pathways, EDCs have been implicated in the development and progression of several health outcomes, particularly reproductive, metabolic, neurodevelopmental, and hormone-related disorders. Of particular concern is their involvement in oncogenesis; throughout tumor progression, EDCs facilitate immune dysregulation and metabolic reprogramming via complex interactions across multiple targets and signaling pathways. Elucidating these underlying regulatory mechanisms is imperative for developing robust preventive strategies. This review synthesizes evidence regarding bisphenols, per- and polyfluoroalkyl substances (PFAS), heavy metals, pesticides, phthalates, polychlorinated biphenyls (PCBs), and other emerging pollutants. We specifically highlight their mechanistic roles across three critical domains: carcinogenesis, immune modulation, and metabolic reprogramming. Given the long latency periods, significant inter-individual variability, and synergistic mixture effects associated with EDC exposure, current risk assessments and causal inferences remain constrained. Future research must integrate high-precision exposure science, prospective longitudinal cohorts, and multi-omic mechanistic validation, while incorporating critical developmental windows and mixture-exposure frameworks. Ultimately, such advancements will provide a more reliable evidence base for exposure prevention, regulatory decision-making, and risk stratification and intervention strategies for exposure-related malignancies.

## Graphical Abstract

**Figure:**
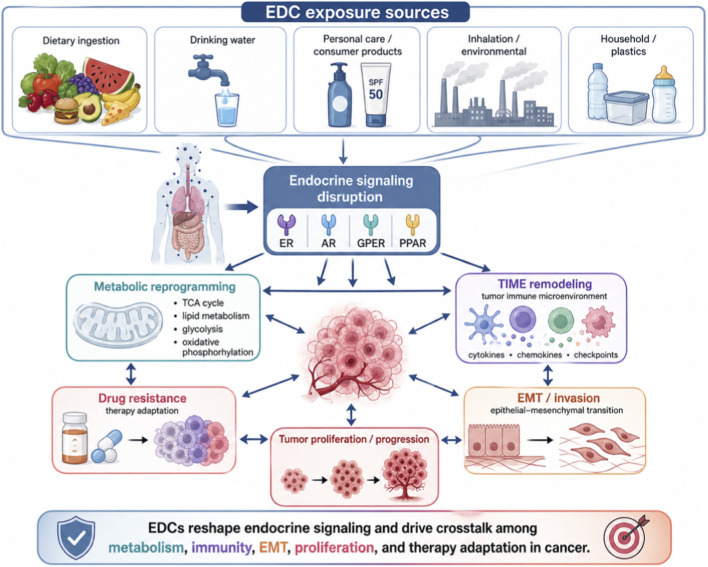

## 1 Introduction

Global cancer burden is accelerating; by 2050, annual incidences are expected to reach 35.3 million, a 76.6% increase from the 2022 estimate of 20.0 million cases (Bizuayehu et al., 2024), although the underlying etiologies remain multifaceted (Hanahan, 2026). Beyond conventional risk factors, such as tobacco use, excessive alcohol consumption, oncogenic infections, and ionizing radiation, metabolic stressors like obesity and diabetes are increasingly recognized (Swanton et al., 2024). Nevertheless, these established factors account for only a fraction of total cancer incidence, underscoring the need to better characterize overlooked environmental contributors to cancer risk.

Exposure to endocrine-disrupting chemicals (EDCs) is estimated to account for 56.52% of all-cause mortality, corresponding to approximately 1.53 million deaths and an economic toll of roughly USD 189.7 billion (Fan et al., 2023). Representative EDCs include bisphenol compounds, particularly bisphenol A (BPA), organochlorine compounds such as dichlorodiphenyltrichloroethane (DDT) and polychlorinated biphenyls (PCBs), polybrominated flame retardants, alkylphenols, and phthalates (Ahn and Jeung, 2023). Crucially, even sub-threshold or low-level exposure to phthalates, polycyclic aromatic hydrocarbons, phytoestrogens, and toxic metals has been linked to substantial healthcare expenditures and societal health burdens. With a substantial proportion of identified EDCs exhibiting tumor-relevant biological activity (Pan et al., 2023; Dou et al., 2026), these chemicals have been implicated in the pathogenesis and progression of hormone-sensitive malignancies, such as ovarian cancer, breast cancer (BC), and prostate cancer (PCa) (Liu et al., 2023; Macedo et al., 2023). However, this figure is likely an underestimate due to the absence of mandatory testing and the long clinical latency of EDC-associated oncogenesis (Modica et al., 2023). Beyond initial carcinogenesis, EDCs may influence tumor evolution by perturbing hormonal signaling, metabolic flux, and the tumor microenvironment (TME), including immune components, thereby contributing to metabolic reprogramming and therapy-related adaptive responses (Lee et al., 2024; Czaczkowska et al., 2025).

Given the escalating socioeconomic burden and complex multi-organ interactions of EDCs (Modica et al., 2023; Feijó et al., 2025), this review aims to move beyond a chemical-by-chemical summary and instead develop an integrated mechanistic framework for understanding EDC-associated cancer-related processes. Unlike previous reviews that have primarily focused on individual EDC classes, hormone-dependent carcinogenesis, or isolated tumor microenvironmental effects, this review emphasizes a unified model linking endocrine signaling disruption, tumor immune microenvironment (TIME) remodeling, metabolic rewiring, epithelial–mesenchymal transition (EMT), tumor progression, and therapy-related adaptation.

Specifically, we synthesize evidence across representative EDC classes, including bisphenols, PFAS, heavy metals, pesticides, phthalates, and PCBs, and identify shared regulatory hubs such as ER/AR/GPER, AhR, PPARs, PI3K–AKT–mTOR, AMPK, NF-κB, ROS, mitochondrial dysfunction, glycolysis, lipid metabolism, and immune suppression. By integrating receptor-level disruption, immune remodeling, and metabolic reprogramming into a single crosstalk-based framework, this review provides a translational perspective for understanding how chronic environmental exposure may influence cancer susceptibility, progression, and therapeutic response.

## 2 EDCs and carcinogenesis

The oncogenic potential of EDCs is primarily manifested in hormone-sensitive tissues, most notably the breast, prostate, testes, ovaries, and thyroid (Ahn and Jeung, 2023; S et al., 2025; Long et al., 2025). Approximately 43%–67% of relevant studies support a positive correlation between EDC exposure and heightened tumorigenic risk; specifically, the strongest association was observed in thyroid cancer (67%), while ovarian cancer showed the lowest proportion of supportive evidence (43%) (Macedo et al., 2023). Collectively, EDCs perturb endocrine homeostasis and orchestrate shifts in cell fate via diverse molecular pathways, thereby fostering a permissive environment for tumor initiation and subsequent progression.

Mechanistically, the estrogen-mimetic properties of EDCs are considered the primary drivers of hormone-dependent malignancies (Lozano-Herrera et al., 2025). BC, now the second most prevalent malignancy worldwide and the fifth leading cause of cancer-related mortality (Kim et al., 2025), has exhibited a rising incidence partly attributable to EDC exposure. However, robust epidemiological and experimental evidence currently exists for only a select group of compounds, including diethylstilbestrol, dichlorodiphenyltrichloroethane (DDT), dioxins, and BPA (Liu et al., 2023; Wan et al., 2022). Similarly, because environmental EDCs can simulate endogenous ligands and activate signaling cascades critical to PCa proliferation, the link between EDC exposure and adverse PCa prognoses has garnered significant scientific scrutiny (Alwadi et al., 2022; Feng et al., 2021; Alwadi et al., 2023).Furthermore, thyroid cancer represents the most prevalent endocrine malignancy, with approximately 567,000 cases reported globally. Notably, it disproportionately affects women, accounting for an estimated 5.1% of the total female cancer burden (Wa et al., 2026). While mechanistic insights into EDC-induced thyroid dysfunction have matured, direct evidence of their definitive carcinogenic impact remains comparatively sparse, necessitating high-fidelity longitudinal studies to elucidate causal relationships and identify the underlying molecular determinants.

The most rigorously characterized estrogenic EDCs include phthalates, PCBs, and BPA (Malik and Mukherjee, 2025). These compounds function by mimicking or amplifying estrogenic stimuli, thereby activating downstream signaling pathways that facilitate tumor cell proliferation, invasion, and metastatic potential (Edlund et al., 2025). Consequently, a mechanistic paradigm has emerged: aberrant hormone receptor activation, accelerated proliferation and invasiveness, elevated oncogenic risk (Akbariani et al., 2025), which is central to our current understanding of EDC-mediated carcinogenesis. However, given the ubiquitous nature of EDCs and the multifaceted complexity of their disruptive mechanisms, sustained evaluation of their long-term carcinogenic impact is essential (Del Río Barrera et al., 2025). Such efforts are critical for identifying high-risk substances, implementing regulatory controls, and enhancing public health strategies to mitigate environmental exposure.

### 2.1 Bisphenols: from BPA to structural analogs

Bisphenol compounds are prototypical EDCs ubiquitous in polycarbonate plastics, epoxy resins, and diverse consumer goods (Xu et al., 2025). BPA remains one of the most high-volume industrial chemicals globally, frequently detected in food-contact materials and household items (Zaborowska et al., 2023). As regulatory scrutiny of BPA’s health risks has intensified, structural analogs, such as bisphenol S (BPS), bisphenol F (BPF), and bisphenol AF (BPAF), have been introduced as substitutes (Xu et al., 2025). However, the assumption of “safety by substitution” is increasingly challenged. Due to their structural similarities, these analogs often mirror or exceed the biological activity of BPA, necessitating a systematic elucidation of their carcinogenic potential.

Notably, BPA has been implicated in the formation of premalignant lesions and malignant foci in the breast and prostate (Al-Ani et al., 2025). In BC research, the “exposure window–tissue microenvironment–susceptibility” framework has expanded our understanding of BPA. Recent evidence suggests that *in utero* exposure increases BC susceptibility by enhancing collagen deposition and extracellular matrix (ECM) density, thereby elevating tissue stiffness (Wormsbaecher et al., 2020). Furthermore, BPA promotes the proliferation of ERα-positive cells (e.g., MCF-7) via endoplasmic reticulum-related pathways and the induction of long noncoding RNAs like HOTAIR (Raju et al., 2023). Similarly, the link between bisphenols exposure and PCa progression has gained significant traction. In the murine prostate, neonatal exposure activates the histone methyltransferase MLL1, inducing persistent H3K4 trimethylation in cancer-related genes, a phenomenon described as an epigenetic “early exposure–long-term memory” effect (Wang et al., 2016). In RWPE-1 cells, BPA induces a transformed phenotype characterized by increased migration and anchorage-independent growth (Caneparo et al., 2024). However, it should be emphasized that most *in vitro* human PCa models lack ER expression and activity, which limits the extrapolation and integration of “estrogen-like” mechanisms. Consequently, further systematic investigation is required to elucidate the pathways through which bisphenols modulate the PCa tumor microenvironment (TME) (Lafront et al., 2020).

Beyond estrogenic activity, bisphenols significantly interfere with androgen receptor (AR) signaling. Using luciferase reporter systems, research has demonstrated that nearly all bisphenol analogs can inhibit AR activity (Grimaldi et al., 2019). Notably, bisphenol mixtures elicit estrogenic and anti-androgenic effects at lower cumulative concentrations than single agents, suggesting that mixed exposures induce more complex endocrine perturbations (Grimaldi et al., 2019). Mechanistically, BPA inhibits dihydrotestosterone (DHT)-induced AR nuclear translocation in a concentration-dependent manner (Huang et al., 2019). In TM4 Sertoli cells, BPA functions as an AR antagonist by disrupting N- and C-terminal interactions and recruiting corepressors such as SMRT and NCoR, thereby suppressing AR-mediated transcription (Wang et al., 2017). This bidirectional disruption of the ER/AR axis likely governs the hormone-dependent progression of PCa (Stanojević and Sollner, 2025). Conversely, the link between BPA and thyroid cancer remains a significant evidence gap, with sparse data available from animal or human studies (Gorini et al., 2020).

Notably, the oncogenic influence of BPA extends beyond classical nuclear receptors into complex, integrated signaling networks. In testicular seminoma (JKT-1) and Sertoli (TM4) cells, BPA drives proliferation by activating cAMP/cGMP-dependent protein kinases and inducing the phosphorylation of ERK and CREB (Bouskine et al., 2009). In BC models, BPA and its analogs upregulate genes associated with invasion and metastasis via ERα-mediated mechanisms (Combarnous and Nguyen, 2022). Furthermore, low-dose BPA triggers the phosphorylation of protein kinase D1 (PKD1), a key driver of anchorage-independent growth (Merzoug-Larabi et al., 2019). In triple-negative BC, BPA enhances oncogenic phenotypes via the GPER–FAK–Src–ERK2 cascade, promoting focal adhesion assembly and cellular invasiveness (Castillo-Sanchez et al., 2020; Donini et al., 2020).

The transition to bisphenol substitutes, such as BPAF, has not necessarily mitigated biological risk. BPAF exhibits a higher binding affinity for ER-related targets than BPA, inducing estrogen-responsive genes through both genomic and nongenomic pathways (e.g., ERα and ERK1/2 activation) (Zhao Q. et al., 2019). BPAF also facilitates crosstalk between the membrane glycoprotein amphiregulin (AREG) and receptor tyrosine kinases, further stimulating the proliferation of ER-positive BC cells (Zhao Q. et al., 2019). Additionally, BPAF triggers GPER-mediated rapid signaling via the ERK and PI3K/Akt pathways. Similarly, BPS has been linked to BC progression by acting on stromal and stem cells (Buoso et al., 2020). While BPA promotes survival following DNA damage and drives adipogenesis via ERR-γ activity, BPS enhances lipid accumulation through ER-mediated mechanisms (Buoso et al., 2020). These divergent yet complementary effects suggest that bisphenols confer a sustained growth advantage by facilitating stress adaptation and metabolic phenotype remodeling. Ultimately, the multi-target impact of these compounds on signal transduction and metabolic flux provides a critical foundation for understanding EDC-driven therapeutic resistance and metabolic reprogramming.

### 2.2 Per- and polyfluoroalkyl substances (PFAS)

PFAS have long garnered scientific scrutiny due to their pervasive risks to human health (Hong et al., 2025). Characterized by exceptional environmental persistence and mobility, these industrial compounds infiltrate the human body primarily through the ingestion of contaminated water and food, as well as inhalation (Gou et al., 2024). This results in chronic, systemic exposure, which is increasingly associated with the pathogenesis of various malignancies (Ferguson et al., 2026; Tanghal et al., 2026).

The epidemiological landscape of PFAS-associated cancer risk is defined by heterogeneity, with outcomes often contingent upon specific exposure windows and chemical profiles. In the Child Health and Development Studies, prenatal exposure to the perfluorooctane sulfonate/perfluorooctanesulfonic acid (PFOS) precursor N-ethyl perfluorooctane sulfonamidoacetic acid (N-EtFOSAA) correlated positively with BC risk in daughters, while prenatal PFOS exposure exhibited a paradoxical protective effect (Cohn et al., 2020). Similarly, the French E3N cohort identified a positive association between postmenopausal BC and PFOS levels (Mancini et al., 2020), while Danish National Birth Cohort data linked early-pregnancy perfluorooctane sulfonamide to increased postpartum BC incidence, despite a potential risk reduction associated with perfluorohexane sulfonate (PFHxS) (Bonefeld-Jørgensen et al., 2014). Additional evidence from Greenland and Italy’s Veneto region corroborates the link between environmental PFAS contamination and elevated BC mortality (Aru et al., 2025). These divergent findings underscore that the timing of exposure, particularly during critical developmental windows, and specific PFAS congeners are pivotal determinants of oncogenesis.

Mechanistically, PFAS promote malignancy through multifaceted pathways. They act as potent endocrine disruptors by targeting PPARγ and ESR1 and facilitate cellular transformation by inducing the epithelial-mesenchymal transition (EMT) and chemoresistance (Yu et al., 2025; Rickard et al., 2025). At the molecular level, PFAS drive invasiveness via epigenetic and autophagic dysregulation, specifically through m6A modification and the activation of MAPK15/autophagy and PI3K–Akt/HIF-1 signaling (Qian et al., 2025). Furthermore, PFAS-induced immunotoxicity, characterized by lymphocyte atrophy and systemic immunosuppression, may severely compromise antitumor surveillance (Du et al., 2025). Notably, while PFOS and perfluorooctanoic acid (PFOA) lack intrinsic estrogenic activity, they function as “signaling sensitizers” that synergistically potentiate the effects of 17β-estradiol, thereby amplifying estrogen-responsive gene expression, ERK1/2 activation, and cellular proliferation in hormone-deprived BC models (Sonthithai et al., 2016). Mechanistically, selected PFAS may influence the TIME by disrupting cytokine signaling, weakening T-cell and NK-cell effector functions, and altering macrophage-associated inflammatory responses. These immune effects may interact with PFAS-induced metabolic stress, particularly mitochondrial dysfunction and lipid dysregulation, thereby creating a tumor-supportive microenvironment that is both metabolically stressed and immunologically suppressed.

Despite the industrial shift toward short-chain substitutes to mitigate bioaccumulation, their high environmental mobility and persistence maintain a significant long-term health burden (Dai et al., 2024). To date, the link between PFAS and gastrointestinal malignancies remains inconclusive, largely due to the limitations of retrospective and cross-sectional study designs (Zhang et al., 2025; Sun H. et al., 2025). Consequently, large-scale prospective cohorts integrated with multi-omic analyses are imperative to elucidate the carcinogenic mechanisms of PFAS and establish a robust scientific framework for environmental health policy and risk assessment.

### 2.3 Heavy metals as endocrine disruptors

Beyond organic compounds, specific heavy metals have emerged as potent environmental EDCs (Jomova et al., 2025). Owing to their environmental persistence and diverse anthropogenic sources, metals such as cadmium (Cd), lead (Pb), and chromium (Cr) facilitate malignancy primarily through endocrine interference and the induction of oxidative stress (Caini et al., 2025; Ali-El-Dein et al., 2025). Their ubiquity is evidenced by their frequent detection in human biological matrices, often tracing back to industrial stabilizers used in plastic manufacturing (Turner and Filella, 2021).

By perturbing hormonal homeostasis, heavy metals may directly or indirectly exacerbate estrogen-dependent pathologies, including polycystic ovary syndrome (PCOS), endometriosis, and endometrial cancer (Laws et al., 2021). In particular, Cd exposure is significantly associated with an increased incidence of endometrial cancer, underscoring the impact of metal-induced endocrine interference on gynecologic cancers (Chitakwa et al., 2024). Furthermore, the oncogenic potential of these metals is inextricably linked to the generation of reactive oxygen species (ROS) (Elbekai and El-Kadi, 2004). Hexavalent chromium [Cr(VI)], a well-established carcinogen, triggers systemic oxidative stress by inducing hydroxyl radicals and superoxides (Pekmezci et al., 2025). A coordinated ROS–AHR axis mediates the oncogenic effects of heavy metals, wherein Cr(VI)-induced oxidative stress triggers oxylipin production to activate Aryl Hydrocarbon Receptor (AHR) signaling. This pathway is similarly upregulated by Cd exposure, leading to the modulation of pro-tumorigenic gene expression (Anwar-Mohamed et al., 2009). In addition to direct oxidative damage, heavy metal-induced ROS and AhR/NF-κB signaling may reshape inflammatory and immune-regulatory networks within the TME. Persistent oxidative stress can promote the production of pro-inflammatory cytokines, including IL-6, TNF-α, and IL-1β, while also favoring immunosuppressive mediators such as IL-10 and TGF-β under chronic exposure conditions. These cytokine changes may contribute to macrophage polarization, impaired cytotoxic lymphocyte activity, and weakened immune surveillance, thereby linking metal-associated oxidative stress to TIME remodeling.

Evidence linking heavy metal exposure to PCa risk is also mounting. Meta-analyses indicate a significant dose-response relationship between arsenic (As) exposure and PCa risk (relative risk (RR) 1.18, 95% confidence interval (CI) 1.06–1.30) (Ahn et al., 2020; Yang et al., 2022). While Cd may not act as a primary mutagenic “initiator” in the prostate, it functions as a potent tumor promoter, with exposure correlating to higher tumor grades and increased invasiveness (Kou et al., 2024). The clinical gravity of these associations is underscored by the 2021 European Association of Urology (EAU) guidelines, which formally recognize Cr, organochlorine pesticides, and asbestos as significant risk factors for PCa (Gandaglia et al., 2021; Krstev and Knutsson, 2019; Dutheil et al., 2020). In summary, heavy metals represent a critical class of EDCs that drive malignancy through a synergy of endocrine disruption, oxidative damage, and aberrant receptor signaling. These mechanisms provide a molecular framework for understanding how environmental exposure contributes to therapeutic resistance and metabolic reprogramming in advanced cancer.

### 2.4 Pesticides

Owing to their environmental persistence and high bioaccumulative potential, pesticides represent a significant threat to global public health (Xie et al., 2024), possessing multi-organ, target-driven carcinogenic potential. While no EDCs have been definitively categorized as primary ovarian carcinogens, accumulating evidence suggests that exposure to dioxins and chlorotriazine herbicides is associated with an elevated risk of ovarian pathologies. *In vitro*, chronic exposure to 2,3,7,8-tetrachlorodibenzo-p-dioxin has been shown to promote the development of ovarian tumors (Davis et al., 2000). Furthermore, case-control studies indicate that women with a history of exposure to relevant EDCs may face a nearly 2.7-fold increase in ovarian tumor risk (Donna et al., 1989). Moreover, systematic investigations into organophosphate pesticides, including chlorpyrifos, parathion-methyl, terbufos, and dichlorvos, consistently report a positive correlation between cumulative exposure and increased BC risk (Lerro et al., 2015; Engel et al., 2017; Tayour et al., 2019). Beyond maternal health, pesticide exposure significantly compromises fetal development, influencing oncogenic outcomes across generations. Maternal exposure during pregnancy is associated with a heightened incidence of testicular germ cell tumors (TGCT) in male offspring (Bräuner et al., 2021). In California, residential proximity to pesticide application prior to birth was linked to increased adolescent TGCT risk (Swartz et al., 2022), a finding corroborated by French studies identifying household fungicide use as a significant risk factor for non-seminomatous TGCT (odds ratio (OR) 1.92; CI 1.12–3.30) (Danjou et al., 2021). This transgenerational risk is further evidenced by a positive correlation between parental occupational exposure and TGCT incidence in the next-generation (Paul et al., 2023). From an immune perspective, pesticide exposure during developmental windows may also influence immune programming, inflammatory tone, and later-life antitumor surveillance. Dioxin-like and organophosphate compounds can interact with AhR- and NF-κB-related pathways, which are closely linked to cytokine production, macrophage activation, and T-cell differentiation. Such immune alterations may not be sufficient to initiate malignancy alone, but they may cooperate with endocrine disruption and developmental programming to create a more permissive tissue microenvironment for hormone-related tumor development. In conclusion, pesticides may influence cancer susceptibility by interfering with hormonal signaling during critical developmental windows. This mechanistic disruption provides a compelling explanation for the rising incidence of hormone-dependent cancers across diverse populations.

### 2.5 Phthalates: ubiquitous plasticizers and multi-target effects

Phthalates represent a major class of environmental EDCs with diverse industrial applications (Yin et al., 2025). Primarily utilized as plasticizers to enhance the flexibility and durability of rigid polymers, such as polyvinyl chloride (PVC), they also serve as solvents or functional additives in non-plasticizing contexts, including cosmetics and personal care products (Tsatsakis et al., 2019; Mariana et al., 2016). Given their pervasive presence in consumer goods and industrial materials, phthalates lead to ubiquitous human exposure, typically following an exposure pattern of chronic, low-dose, and multi-compound “mixtures,” which may persistently perturb endocrine homeostasis and systemic metabolism.

Phthalates have been implicated in tumor-related processes through potential effects on metabolic remodeling and endocrine–microenvironmental interactions. The link between phthalates and PCa risk appears to be modulated by the host’s metabolic landscape (Jiang et al., 2025). For instance, a significant association between phthalate exposure and PCa risk was identified specifically among obese men in Taiwan, suggesting a synergistic interaction between these chemicals and metabolic dysfunction (Chuang et al., 2020). Emerging evidence also suggests that maternal serum levels of phthalate metabolites are associated with dysregulated lipid metabolism, indicating that exposure during critical windows may exert long-term systemic effects (Zhou et al., 2018). In BC models, both dibutyl phthalate (DBP) and butyl benzyl phthalate (BBP) have been shown to accelerate the growth of MDA-MB-231 xenograft tumors (Hsieh et al., 2012). Additionally, phthalate exposure is consistently associated with an increased incidence of BC, highlighting the distinct vulnerability of endocrine-responsive tissues to chemical perturbations (Wan et al., 2022).

### 2.6 PCBs: persistent metabolic and endocrine disruptors

PCBs, a class of synthetic organohalogen compounds once valued for their chemical stability and electrical insulation, remain critical public health threats despite a 1979 production ban (Christensen et al., 2021). Their environmental persistence and bioaccumulative nature facilitate chronic human exposure via the food chain. Beyond classical endocrine disruption, PCBs may interfere with cellular metabolic networks, acting as exogenous disruptors of lipid homeostasis and fatty acid biosynthesis (Li et al., 2020).

High environmental chemical burdens are associated with adverse health outcomes, as evidenced by the elevated incidence of differentiated thyroid cancer in Staten Island, which coincides with high environmental burdens of PCBs (Van Gerwen et al., 2021). The impact of PCBs on reproductive malignancies is particularly evident in transgenerational studies. Maternal serum levels of organohalogen compounds, including specific PCB congeners, are significantly associated with a heightened risk of TGCT in male offspring (hazard ratio (HR) 2.53; OR 2.4) (Bräuner et al., 2021). Notably, exposure to estrogenic PCB congeners correlates with increased risk of both seminomatous and non-seminomatous tumors (OR 2.5), suggesting that PCBs elevate cancer risk through the synergistic effects of endocrine disruption and developmental programming (Cheng et al., 2021a; Cheng et al., 2021b). Moreover, PCB metabolites, such as PCB29-pQ, have been reported to promote tumor-relevant metabolic phenotypes through a coordinated “glycolytic reprogramming–nutrient uptake–metastasis” axis, characterized by enhanced aerobic glycolysis and GLUT1 upregulation (Qin et al., 2022). This local metabolic adaptation is further augmented by systemic effects; PCBs induce metabolic disorders, including obesity and type 2 diabetes, which alter nutrient availability and pro-inflammatory signaling within the TME(103). Consequently, PCBs may contribute to cancer-related processes through both systemic metabolic disruption and local modulation of tumor metabolism.

In summary, PCBs drive malignancy through a multifaceted synergy of metabolic reprogramming, systemic metabolic dysregulation, and developmental programming, providing a comprehensive mechanistic basis for their long-term carcinogenic potential. These metabolic disturbances may further influence immune composition and function within the TIME. For example, dysregulated lipid availability and chronic low-grade inflammation can favor tumor-associated macrophage-like phenotypes, suppress cytotoxic T-cell activity, and impair NK-cell-mediated immune surveillance. In parallel, PCB-associated activation of AhR, oxidative stress, and inflammatory signaling may contribute to cytokine imbalance and checkpoint-related immunosuppressive states, although direct clinical evidence linking PCB exposure to immune checkpoint therapy response remains limited.

## 3 EDCs and the acquisition of therapeutic resistance

Clinical failure in oncology is frequently associated with the emergence of therapy-resistant phenotypes under selective treatment pressure (Chen et al., 2025). Although therapeutic resistance has traditionally been attributed to intrinsic genomic instability, clonal evolution, drug efflux, and adaptive survival signaling, emerging evidence suggests that exogenous environmental exposures may also influence cellular states relevant to treatment response (Lagunas-Rangel, 2026). In the context of EDCs, these effects should be interpreted cautiously, as direct clinical evidence remains limited and varies by compound class, cancer type, exposure window, and therapeutic context. Nevertheless, selected EDCs have been reported to modulate stress adaptation, programmed cell death, epithelial–mesenchymal transition (EMT), mitochondrial function, receptor-mediated survival signaling, and metabolic plasticity, thereby potentially creating biological conditions that favor therapy-related adaptation (Lagunas-Rangel, 2026; Cortes-Ramirez et al., 2024).

PFAS provide one of the clearest experimental examples linking EDC exposure to chemoresistance-related phenotypes. Although PFAS exposure has been associated with adverse outcomes in gynecologic cancer-related settings, the causal mechanisms linking PFAS to clinical chemoresistance remain incompletely defined (Cortes-Ramirez et al., 2024). Recent investigations by Rickard et al. demonstrated that combined PFAS exposure enhanced cell survival in human ovarian cancer models following carboplatin treatment (Rickard et al., 2022). This phenotype was accompanied by marked mitochondrial dysfunction, suggesting that PFAS may contribute to platinum-tolerant cellular states by altering mitochondria-mediated energy metabolism and increasing the apoptotic threshold (Rickard et al., 2022). In addition, selected PFAS have been linked in experimental models to EMT, chemoresistance-associated cellular phenotypes, autophagic dysregulation, m6A modification, and PI3K–Akt/HIF-1 signaling (Yu et al., 2025; Rickard et al., 2025; Qian et al., 2025). These findings suggest that PFAS may influence therapy-related adaptation through the convergence of mitochondrial stress, altered programmed cell death, metabolic remodeling, and EMT-associated plasticity, although validation in longitudinal clinical cohorts remains necessary.

Bisphenol compounds may also contribute to therapy-related adaptive phenotypes through receptor-mediated signaling, tumor-suppressor disruption, and metabolic remodeling. BPA can engage a multi-receptor network, including ERα, ERβ, GPER, membrane-associated ERs, thyroid hormone receptors, and AR (Vandenberg et al., 2009). In cancer-related models, BPA and its analogs have been reported to activate downstream pathways such as ERK1/2, PI3K–Akt, PKD1, and GPER–FAK–Src–ERK signaling, which are associated with proliferation, migration, anchorage-independent growth, and invasive phenotypes (Merzoug-Larabi et al., 2019; Castillo-Sanchez et al., 2020; Donini et al., 2020; Zhao Q. et al., 2019). Importantly, BPA exposure has been associated with p53 inactivation in human cancer cells, potentially weakening DNA damage responses and apoptosis-related tumor-suppressive mechanisms (Jiang et al., 2025; Chuang et al., 2020; Zhou et al., 2018). In BC models, dibutyl phthalate (DBP) and butyl benzyl phthalate (BBP) have been shown to accelerate MDA-MB-231 xenograft tumor growth, suggesting that phthalates can support aggressive tumor behavior under experimental conditions (Hsieh et al., 2012). Although these observations do not directly establish phthalate-induced treatment resistance, they suggest that phthalate-associated metabolic and proliferative changes may be relevant to tumor-cell stress adaptation.

Compared with phthalates, PCBs provide a more direct mechanistic link between metabolic rewiring, EMT, and aggressive tumor phenotypes. The quinone-type PCB metabolite PCB29-pQ has been reported to induce GLUT1 expression and activate the GLUT1/integrin β1/Src/FAK signaling cascade, thereby promoting EMT, extravasation, and metastatic dissemination (Qin et al., 2022; Zhao H. et al., 2019). Since EMT and altered glucose uptake are frequently associated with adaptive survival states in cancer, PCB29-pQ-associated glycolytic reprogramming and EMT-like transition may represent a plausible route through which persistent pollutants contribute to therapy-related phenotypes. Beyond local tumor effects, PCBs have also been associated with systemic metabolic disorders, including obesity and type 2 diabetes, as well as altered fatty acid biosynthesis and activation pathways, particularly under mixed-exposure conditions involving other EDCs such as DDT and PFAS(97, 103). These systemic metabolic disturbances may further shape a tumor-supportive environment that affects nutrient availability, inflammatory signaling, and stress tolerance.

AhR-related signaling and oxidative stress may serve as additional mechanistic bridges linking EDC exposure to therapy-related adaptation. Heavy metals and dioxin-like compounds can induce oxidative stress and activate ROS-associated signaling networks (Elbekai and El-Kadi, 2004; Pekmezci et al., 2025; Anwar-Mohamed et al., 2009). In particular, Cr(VI)-induced oxidative stress may trigger oxylipin production and subsequent AhR activation, while Cd exposure has also been reported to upregulate AhR-related pathways and modulate pro-tumorigenic gene expression (Anwar-Mohamed et al., 2009). These ROS–AhR-related effects may interact with inflammatory signaling, metabolic remodeling, and apoptosis-related pathways, thereby contributing to cellular states that are more tolerant of stress. However, direct evidence linking EDC-induced AhR activation to clinically established therapeutic resistance remains limited and should be regarded as mechanistically plausible rather than definitive.

Overall, EDC-associated therapeutic resistance is best understood as a multi-layered adaptive framework rather than a single linear pathway. Based on the available evidence, selected EDCs may influence therapy-related phenotypes through several interconnected mechanisms: disruption of DNA damage responses and p53-mediated apoptosis; mitochondrial dysfunction and increased apoptotic threshold; activation of receptor-mediated survival pathways such as ERK, PI3K–Akt, PKD1, and FAK/Src signaling; EMT-associated plasticity and invasive transition; and metabolic adaptation involving glycolysis, lipid metabolism, nucleotide biosynthesis, oxidative phosphorylation, and redox homeostasis (Merzoug-Larabi et al., 2019; Castillo-Sanchez et al., 2020; Donini et al., 2020; Zhao Q. et al., 2019; Yu et al., 2025; Rickard et al., 2025; Qian et al., 2025; Qin et al., 2022; Rickard et al., 2022; Zhao H. et al., 2019; Zhao et al., 2022; Liu et al., 2020; Ma et al., 2021; Huang et al., 2020; Zhao C. et al., 2019; Dairkee et al., 2012). These mechanisms provide a plausible basis for linking endocrine disruption, metabolic rewiring, and therapy-related adaptation. Future studies should integrate exposure assessment, treatment-response data, patient-derived models, and multi-omic profiling to determine whether EDC-associated molecular signatures can predict therapeutic response, recurrence risk, or resistance-associated tumor phenotypes in clinically relevant settings.

## 4 Integrated multi-target and multi-pathway effects of EDCs on metabolic reprogramming

In tandem with phenotypic switching, tumor cells undergo profound metabolic reprogramming to satisfy the bioenergetic and biosynthetic demands of rapid proliferation (Cordani et al., 2024). Metabolic hubs, specifically the PI3K/Akt/mTOR and AMPK pathways, serve as master regulators of metabolic flux. By modulating the expression and catalytic activity of rate-limiting enzymes, these pathways remodel cellular networks across glucose, lipid, and amino acid metabolism (Tan et al., 2022; Talwar et al., 2023; Ambrosini et al., 2020; Hoxhaj and Manning, 2020). This metabolic plasticity not only supports survival under conditions of hypoxia and nutrient scarcity but also provides the energetic basis for sustained invasion and the acquisition of therapeutic resistance.

Within the integrated “EMT–metabolic plasticity” framework, the impact of environmental EDCs manifests as a multi-target and multi-pathway modulation of cellular signaling. EDCs exert toxicity by perturbing hormone synthesis, secretion, and receptor-mediated activity, thereby altering the developmental microenvironment of reproductive and other endocrine-sensitive tissues (Yilmaz et al., 2020). Furthermore, EDCs exert a dual influence on the metabolic ecosystem: systemic metabolic disruption, inducing organism-wide disturbances, such as dysregulated lipid/glucose homeostasis and chronic low-grade inflammation; local tumor remodeling, triggering adaptive metabolic shifts and signaling crosstalk within the tumor and its immediate microenvironment. This “dual-route” mechanism, coupling systemic metabolic dysfunction with localized cellular reprogramming, may contribute to tumor-promoting metabolic states and therapy-related adaptive responses.

Importantly, EDC-associated metabolic reprogramming is closely intertwined with immune remodeling. Altered glycolysis, lipid metabolism, mitochondrial function, and redox balance can modify nutrient availability and inflammatory signaling within the TIME. These changes may suppress cytotoxic CD8^+^ T-cell and NK-cell activity, favor macrophage polarization toward tumor-supportive phenotypes, and promote cytokine networks characterized by IL-6, TNF-α, IL-10, TGF-β, CCL2, and CXCL-family chemokines. In addition, activation of AhR, NF-κB, PI3K–AKT–mTOR, HIF-1α, and ROS-related pathways may contribute to immune checkpoint regulation, including PD-1/PD-L1-associated suppressive signaling. Therefore, EDC-induced endocrine and metabolic perturbations should be interpreted together with TIME remodeling rather than as isolated processes (Dairkee et al., 2012). Since p53 is closely involved in treatment-induced apoptosis and genomic stress responses, BPA-associated p53 disruption may represent a plausible mechanism through which bisphenols influence treatment sensitivity. In parallel, BPA and related bisphenol analogs have been shown to alter nucleotide metabolism, purine and pyrimidine pathways, lipid metabolism, glycolytic intermediates, and glutathione metabolism (Zhao et al., 2022; Liu et al., 2020; Ma et al., 2021; Huang et al., 2020; Zhao C. et al., 2019). These metabolic alterations may provide additional support for proliferating or stressed tumor cells under therapeutic pressure.

Phthalates and PCBs may influence resistance-related biology primarily through metabolic remodeling and EMT-associated phenotypes, although direct clinical evidence remains limited. Phthalate exposure has been linked to endocrine–metabolic disturbance, lipid metabolic dysregulation, and metabolic contexts that may modify cancer risk, particularly in metabolically vulnerable populations.

### 4.1 Bisphenol compounds: orchestrating systemic and local metabolic reprogramming

BPA disrupts systemic metabolic homeostasis primarily by targeting the liver, a central hub for energy balance (Winz et al., 2023). Both BPA and 17β-estradiol modulate the urea and tricarboxylic acid (TCA) cycles, resulting in the elevation of intracellular metabolites such as arginine, creatine, and glutamine (Cabaton et al., 2018). Given that the upregulation of amino acid transport and metabolism, particularly of arginine and glutamine, is a hallmark of malignancies providing essential substrates for nucleotide synthesis and redox maintenance, these BPA-associated metabolic shifts may contribute to a systemic environment that favors tumor-related metabolic adaptation (Wei et al., 2020). Beyond amino acids, BPA perturbs lipid metabolism by altering apolipoprotein profiles (Cabaton et al., 2018). In CD-1 mice, hepatic BPA exposure is associated with the upregulation of Apob and the downregulation of Apoc3 (Lin et al., 2017). Collectively, this simultaneous disruption of lipid and amino acid pathways suggests that BPA establishes a foundational systemic context that supports tumor initiation and subsequent local adaptation.

BPA can directly reshape the metabolome within tumor cells. In ER-positive MCF-7 BC cells, BPA exposure significantly accelerates nucleotide metabolism, evidenced by increased levels of precursors such as cytidine triphosphate and uridine diphosphate glucuronic acid (Zhao et al., 2022). Research utilizing a “leave-one-out” mixture analysis confirmed that BPA is a primary driver of purine and pyrimidine metabolic alterations (Liu et al., 2020). Because rapidly proliferating cells require an augmented supply of nucleotides for DNA synthesis, this BPA-mediated enhancement of salvage pathways likely provides the metabolic support necessary to maintain high proliferative rates (Ma et al., 2021). Other bisphenol analogs also demonstrate pro-tumorigenic metabolic effects: BPS disrupts the TCA cycle, purine, and lipid metabolism in MCF-10A cells (Huang et al., 2020); TBBPA/TCBPA alter glycolytic intermediates and glutathione metabolism, perturbing cellular antioxidant systems (Zhao C. et al., 2019); BPF induces shifts in glycerophospholipid degradation and glycolysis-related pathways *in vivo* (Zhao et al., 2018).

The molecular basis of these phenotypic changes resides in the ability of BPA to engage a multi-receptor network, including ERα, ERβ, GPER, membrane-associated ERs, thyroid hormone receptors, and the AR(109). For example, BPA activation of ERβ-mediated ion flux has been proposed as one of the key bases for reduced insulin secretion in pancreatic β cells following BPA exposure (Villar-Pazos et al., 2017). Notably, BPA is also implicated in the acquisition of therapeutic resistance. Evidence suggests that BPA exposure is associated with the inactivation of p53 in human cancer cells, potentially weakening DNA damage responses and apoptotic pathways (Dairkee et al., 2012). By coupling systemic lipid/amino acid disruption with local nucleotide synthesis and the suppression of tumor-suppressor pathways, bisphenols may influence cancer-related metabolic phenotypes and altered treatment sensitivity under specific biological contexts.

### 4.2 PCBs: coupling glycolytic flux with metastatic phenotypes

Direct evidence linking PCBs to tumor metabolic reprogramming remains an emerging field. The quinone-type PCB metabolite, PCB29-pQ, significantly induces the expression of the glucose transporter GLUT1, triggering the GLUT1/integrin β1/Src/FAK signaling cascade, which promotes EMT, extravasation, and metastatic dissemination (Zhao H. et al., 2019). Moreover, inhibiting GLUT1 effectively attenuates the pro-metastatic effects of PCB29-pQ *In vivo*, underscoring a functional cascade where PCBs remodel nutrient uptake and energy metabolism to confer a migratory and invasive advantage to tumor cells (Qin et al., 2022).

Beyond their localized effects within the tumor, PCBs function as systemic metabolic disruptors. They contribute to oncogenic risk by inducing lipid metabolic disorders and promoting chronic conditions such as obesity and type 2 diabetes (Shan et al., 2020). Metabolomic and pathway enrichment analyses reveal that elevated blood PCB concentrations correlate with significant alterations in fatty acid biosynthesis and activation pathways. Notably, these perturbations are amplified when PCB exposure occurs in conjunction with other EDCs, such as DDT and PFAS. This suggests that mixed-exposure scenarios exacerbate disruptions within lipid metabolic networks (Li et al., 2020). Given that these lipophilic EDCs interact with nuclear transcription factors to regulate fatty acid uptake and biosynthesis, their dual roles as “systemic metabolic disruptors” and “local tumor metabolic promoters” likely act additively to foster a pro-carcinogenic environment (Shan et al., 2020).

At the population level, EDCs promote metabolic abnormalities by activating shared downstream pathways, which in turn serve as major risk factors for hormone-dependent malignancies. For example, dyslipidemia, a hallmark of systemic metabolic disturbance, provides a pro-inflammatory and nutrient-rich milieu that supports the development of prostate, colon, and pancreatic cancers (Winz et al., 2023). In the context of BC, altered lipid metabolism and elevated circulating free fatty acids have been directly correlated with increased incidence and enhanced tumorigenicity (Marino et al., 2020; Madak-Erdogan et al., 2019). Crucially, the exposure window is a decisive factor: EDC exposure during sensitive developmental periods, such as *in utero* and lactation, can “early-program” the metabolome and mammary architecture. This programming may leave adult mammary tissue more susceptible to malignant transformation by optimizing metabolic supply and hormonal responsiveness for future tumor growth (Winz et al., 2023). In summary, PCB-related research highlights a multifaceted oncogenic model where PCBs couple intra-tumoral metabolic remodeling with systemic dysregulation, particularly when exposure occurs during critical developmental windows.

### 4.3 Convergent mechanistic hubs linking EDC exposure to cancer-related phenotypes

Although different classes of EDCs vary substantially in chemical structure, exposure route, persistence, and tissue distribution, accumulating evidence indicates that their cancer-related effects may converge on a set of shared molecular and cellular hubs. These hubs provide a mechanistic bridge between chemical-specific observations and broader cancer phenotypes, including endocrine dysregulation, metabolic reprogramming, immune remodeling, epithelial–mesenchymal transition (EMT), tumor progression, and therapy-related adaptation.

At the receptor level, several EDCs interfere with nuclear and membrane-associated hormone signaling pathways, particularly estrogen receptors (ERs), androgen receptor (AR), G protein-coupled estrogen receptor (GPER), aryl hydrocarbon receptor (AhR), and peroxisome proliferator-activated receptors (PPARs). Bisphenols primarily affect ER-, AR-, and GPER-mediated signaling, whereas dioxins, PCBs, and heavy metals are more closely associated with AhR activation and oxidative stress-related transcriptional responses. PFAS, phthalates, and PCBs may also interact with PPAR-related pathways, thereby linking endocrine disruption to lipid metabolism, adipogenesis, and systemic metabolic dysfunction. These receptor-level perturbations can further propagate to downstream signaling cascades, including PI3K–AKT–mTOR, AMPK, MAPK, and NF-κB pathways.

Beyond receptor signaling, oxidative stress and mitochondrial dysfunction represent important convergent mechanisms across multiple EDC classes. Reactive oxygen species (ROS) generation may contribute to DNA damage responses, inflammatory signaling, metabolic rewiring, and apoptosis resistance. In parallel, altered mitochondrial function may shift cellular energy metabolism toward glycolysis, lipid metabolic remodeling, and adaptive survival programs. These metabolic changes can influence not only tumor-cell proliferation but also the tumor immune microenvironment (TIME), as nutrient competition, altered metabolite availability, and inflammatory mediators may collectively support immune suppression and impaired antitumor surveillance.

Importantly, these mechanisms should not be viewed as isolated linear pathways. Instead, EDC-associated endocrine disruption, oxidative stress, metabolic reprogramming, EMT, immune remodeling, and therapeutic adaptation are likely to form an interconnected network. For example, ER/GPER and PI3K–AKT signaling may promote proliferative and survival phenotypes; AhR and NF-κB activation may link environmental exposure to inflammatory and immunosuppressive responses; AMPK and mitochondrial dysfunction may reshape energy metabolism under stress conditions; and EMT may further interact with metabolic plasticity and drug resistance. Therefore, the carcinogenic relevance of EDCs may be better understood through a multi-hub framework rather than through single-compound or single-pathway models.

To clarify these shared mechanisms, we summarized the major convergent molecular hubs across representative EDC classes in Table 1. This integrated framework highlights how diverse environmental exposures may influence cancer-related phenotypes through overlapping receptor, metabolic, inflammatory, and immune-regulatory pathways. We summarized the major exposure sources, cancer associations, receptor and signaling hubs, immune-related effects, and metabolic alterations for representative EDC classes in Table 2. This integrated summary highlights that structurally diverse EDCs do not act through completely isolated mechanisms. Instead, they frequently converge on shared endocrine, inflammatory, immune-regulatory, and metabolic pathways, including ER/AR/GPER, AhR, PPARs, PI3K–AKT–mTOR, AMPK, NF-κB, ROS, mitochondrial dysfunction, glycolysis, lipid metabolism, EMT, and immune suppression.

**TABLE 1:** Shared mechanistic hubs linking EDC exposure to cancer-related phenotypes.

Mechanistic hub	Representative EDC classes	Major downstream effects	Cancer-related relevance
ER/AR/GPER signaling	Bisphenols, phthalates, organochlorine pesticides, PCBs	Hormone receptor activation or antagonism; altered proliferative signaling	Hormone-sensitive tumor progression, altered endocrine responsiveness
AhR signaling	Dioxins, PCBs, heavy metals	Xenobiotic response, inflammatory signaling, oxidative stress-related transcription	Tumor-promoting inflammation, altered immune and metabolic responses
PPARs	PFAS, phthalates, PCBs	Lipid metabolism, adipogenesis, metabolic homeostasis	Obesity-related cancer risk, lipid metabolic remodeling
PI3K–AKT–mTOR	PFAS, bisphenols, PCBs	Cell survival, protein synthesis, glycolytic activation	Proliferation, survival advantage, therapy-related adaptation
AMPK signaling	Bisphenols, PFAS, metabolic EDC mixtures	Energy stress response, mitochondrial regulation, metabolic flexibility	Adaptation to nutrient stress and metabolic plasticity
NF-κB signaling	Heavy metals, pesticides, PCBs, dioxins	Inflammatory cytokines, survival signaling, immune modulation	Chronic inflammation, immune suppression, tumor-promoting microenvironment
ROS and oxidative stress	Heavy metals, pesticides, PCBs, bisphenols	DNA damage, mitochondrial stress, redox imbalance	Genomic instability, apoptosis resistance, inflammatory activation
Mitochondrial dysfunction	PFAS, bisphenols, heavy metals	Altered oxidative phosphorylation, apoptotic threshold, energy imbalance	Drug tolerance, metabolic adaptation, cell survival
Glycolysis	PCBs, bisphenols, PFAS	Increased glucose uptake, GLUT1 regulation, glycolytic flux	Tumor growth, invasion, metabolic reprogramming
Lipid metabolism	PCBs, PFAS, phthalates, bisphenols	Fatty acid biosynthesis, lipid accumulation, adipokine imbalance	Systemic metabolic dysfunction and tumor metabolic support
EMT	PFAS, PCBs, bisphenols, pesticides	Loss of epithelial traits, increased migration and invasion	Metastasis, invasiveness, drug resistance
Immune suppression/TIME remodeling	PFAS, dioxins, PCBs, heavy metals	Cytokine imbalance, lymphocyte dysfunction, checkpoint-related changes	Reduced antitumor surveillance and immune escape

**TABLE 2:** Summary of major EDC classes, exposure sources, cancer associations, mechanistic hubs, immune/metabolic effects, and evidence levels.

EDC class	Major exposure sources	Cancer types mainly implicated	Key receptors/ Pathways	Immune-related effects	Metabolic effects
Bisphenols including BPA, BPS, BPF, BPAF	Polycarbonate plastics, epoxy resins, food-contact materials, thermal paper, consumer products	Breast cancer, prostate cancer, testicular tumors; thyroid cancer evidence remains limited	ERα/ERβ, AR, GPER, ERR-γ, ERK1/2, PI3K–AKT, PKD1, p53-related pathways	Potential modulation of inflammatory signaling and tumor microenvironmental responses; direct immune evidence remains limited	Altered TCA cycle, urea cycle, amino acid metabolism, nucleotide metabolism, lipid metabolism, glycolysis, and glutathione metabolism
PFAS including PFOS, PFOA, PFHxS and related precursors	Contaminated drinking water, food, food packaging, industrial emissions, firefighting foams, household products	Breast cancer, ovarian cancer, kidney/testicular cancer in broader literature; gastrointestinal cancer evidence remains inconclusive	PPARs, ESR1, PI3K–AKT/HIF-1, MAPK15/autophagy, m6A-related pathways, ERK1/2 sensitization	Lymphocyte dysfunction, immunotoxicity, systemic immunosuppression, impaired antitumor surveillance	Mitochondrial dysfunction, altered energy metabolism, lipid dysregulation, metabolic adaptation, possible platinum tolerance
Heavy metals including Cd, Pb, Cr, As	Industrial emissions, contaminated food and water, occupational exposure, pigments/stabilizers, polluted soil and air	Endometrial cancer, prostate cancer, hormone-related malignancies; Cr(VI) is a recognized carcinogenic exposure	ER-related signaling, AhR, ROS-related pathways, NF-κB, oxidative stress response, DNA damage pathways	Chronic inflammatory activation, oxidative-stress-associated immune perturbation, possible impairment of antitumor surveillance	ROS generation, mitochondrial stress, redox imbalance, altered lipid and glucose homeostasis
Pesticides and dioxin-like compounds including organophosphates, chlorotriazines, dioxins	Agricultural use, occupational exposure, contaminated food, residential pesticide application, household fungicides	Breast cancer, ovarian pathologies, testicular germ cell tumors, hormone-related cancers	ER/AR disruption, AhR activation, oxidative stress, inflammatory signaling, developmental programming pathways	Immune modulation through AhR and inflammatory cytokine pathways; possible effects on developmental immune programming	Altered lipid metabolism, oxidative stress, mitochondrial perturbation, endocrine-metabolic disruption during developmental windows
Phthalates including DBP, BBP, DEHP metabolites	PVC plastics, cosmetics, personal care products, food packaging, indoor dust, medical materials	Breast cancer, prostate cancer; risk may be modified by obesity and metabolic status	ER/AR-related signaling, PPARs, metabolic nuclear receptors, inflammatory pathways	Potential modulation of inflammatory and endocrine-immune interactions; direct TIME evidence remains limited	Lipid dysregulation, adipogenesis, altered glucose/lipid metabolism, metabolic dysfunction during pregnancy or early life
PCBs	Legacy industrial pollutants, contaminated fish/meat/dairy products, environmental reservoirs, occupational exposure	Thyroid cancer, breast cancer, testicular germ cell tumors, hormone-related malignancies	AhR, ER-related pathways, PPARs, GLUT1/integrin β1/Src/FAK, NF-κB, oxidative stress pathways	Immune suppression, inflammatory signaling, altered cytokine networks, potential impairment of antitumor immunity	Glycolytic reprogramming, GLUT1 upregulation, fatty acid biosynthesis, lipid metabolic disorders, obesity/type 2 diabetes-related metabolic risk

## 5 Discussion

The health hazards associated with EDCs are characterized by significant latency periods and insidious physiological effects (De Paula Nunes et al., 2026). Physiological shifts triggered during critical early-life developmental windows can permanently alter susceptibility to non-communicable diseases (NCDs), which have reached unprecedented prevalence globally (Organization, 2014). The four primary NCD categories, cancer, cardiovascular diseases, chronic respiratory diseases, and diabetes, account for approximately 36 million annual deaths, disproportionately affecting low- and middle-income countries (Gore et al., 2015). There is increasing scientific consensus that chronic, lifelong exposure to low-dose EDCs contributes substantially to this global burden by “programming” disease risk that manifests only in adulthood. We further organized the evidence into a convergent mechanistic framework. This framework emphasizes that structurally diverse EDCs may affect cancer-related phenotypes through shared receptor, metabolic, inflammatory, and immune-regulatory hubs rather than through completely independent pathways. A central contribution of this review is the integration of diverse EDC-related findings into a shared mechanistic framework. Rather than treating bisphenols, PFAS, heavy metals, pesticides, phthalates, and PCBs as independent toxicological categories, we highlight their convergence on common receptor, inflammatory, immune-regulatory, and metabolic hubs. This scope distinguishes the present review from previous EDC–cancer reviews by emphasizing the crosstalk among endocrine signaling disruption, TIME remodeling, metabolic rewiring, EMT, tumor proliferation, and therapy-related adaptation. Such an integrated perspective is particularly important because real-world exposure is chronic, low-dose, and mixture-based, making single-compound or single-pathway models insufficient to capture the biological complexity of EDC-associated cancer-related processes.

In real-world scenarios, exposure rarely involves isolated agents; instead, humans encounter complex chemical “cocktails.” Long-term exposure to mixtures of persistent organic pollutants (POPs) can elicit cumulative, additive, or synergistic actions that significantly amplify pathological risks (Lauretta et al., 2019). The rising incidence of hormone-related malignancies has intensified scrutiny of EDCs. Some toxicological screening profiles suggest that up to 80% of identified EDCs may display tumor-relevant biological activities; however, this does not necessarily indicate definitive carcinogenicity in humans (Sung et al., 2021; Pierozan et al., 2020; Rocha et al., 2021). Despite rigorous oversight by agencies such as the U.S. U.S. Environmental Protection Agency (EPA) and the Food and Drug Administration (FDA), the environmental persistence of legacy contaminants ensures they remain a developmental threat decades after their official phase-out (Houston and Ghosh, 2020). This necessitates regulatory frameworks that address not only contemporary chemical usage but also the risks posed by environmental persistence.

Three exposure-related dimensions should be emphasized when interpreting EDC-associated cancer-related effects: mixture exposure, low-dose non-monotonic responses, and critical developmental windows. First, real-world EDC exposure rarely involves isolated compounds. Instead, humans are exposed to complex chemical mixtures, particularly persistent organic pollutants, which may exert cumulative, additive, or synergistic biological actions (Lauretta et al., 2019). This mixture-based exposure pattern may partly explain why single-compound studies cannot fully capture the biological complexity of environmental contaminants. Second, the conventional toxicological paradigm based on high-dose linear responses is increasingly challenged by evidence that some EDCs can perturb endocrine and metabolic homeostasis at picomolar or nanomolar concentrations and may produce non-monotonic dose–response patterns (Xu et al., 2025). Therefore, effects observed at low doses cannot always be predicted from high-dose models. Third, exposure timing is a critical determinant of long-term biological consequences. Evidence from bisphenol, PFAS, pesticide, and PCB-related studies suggests that prenatal, neonatal, pregnancy-related, and lactational exposure windows may influence tissue architecture, endocrine responsiveness, metabolic programming, and later cancer susceptibility (Wormsbaecher et al., 2020; Wang et al., 2016; Cohn et al., 2020; Mancini et al., 2020; Bonefeld-Jørgensen et al., 2014; Bräuner et al., 2021; Swartz et al., 2022; Danjou et al., 2021; Paul et al., 2023; Winz et al., 2023). Together, these features highlight the need for future studies to incorporate mixture-based exposure assessment, repeated biomonitoring, exposure-window stratification, and non-linear dose–response modeling.

Assessing EDC risk is further complicated by prolonged biological half-lives and extensive latency periods, which can exceed 50 years between initial exposure and clinical manifestation (Yilmaz et al., 2020). Due to their lipophilic nature, EDCs readily accumulate in white adipose tissue (Francis et al., 2021). Biomonitoring studies have detected these compounds in virtually all human biospecimens, including blood, urine, placenta, and fetal cord blood, highlighting the ubiquity of exposure across the life course (Sabuz Vidal et al., 2021). However, individual phenotypic responses to these stressors vary markedly, influenced by a complex interplay of genetic predisposition, occupational factors, underlying comorbidities, and dietary habits.

The traditional toxicological paradigm, stating that adverse effects occur only at high doses, increasingly challenged by evidence showing that EDCs can exert profound homeostatic disruption even at picomolar or nanomolar concentrations (Xu et al., 2025). The combined influence of low-dose, chronic, and multi-chemical exposure introduces a level of complexity that traditional single-pollutant models cannot capture (Yilmaz et al., 2020). While preclinical studies have elucidated estrogen receptor-mediated pathways, emerging research emphasizes that EDC-driven genomic instability and epigenetic modifications are equally critical drivers of oncogenesis (Sun K. et al., 2025).

To strengthen causal inference in future research, it is imperative to integrate high-precision exposure measurement, longitudinal cohort designs, and multi-omics-based mechanistic validation. Furthermore, practical strategies for exposure mitigation must prioritize vulnerable populations. Evidence indicates higher EDC burdens among low-income communities and occupationally exposed groups (e.g., workers in agriculture, industrial manufacturing, and painting) (Malits et al., 2022). Targeted risk communication, enhanced protective protocols, and the reduction of unnecessary environmental contact are essential to mitigate health inequities. Ultimately, addressing the global EDC threat requires multicenter population studies and standardized analytical methodologies to ensure data comparability. Integrating mixed-exposure frameworks into public health strategies and regulatory policies is paramount, as the chemical mixtures may better reflect real-world exposure conditions and may contribute to EDC-associated disease burden.

Another important implication of this integrated framework is that EDC-associated cancer-related effects may be mediated not only by direct endocrine or metabolic disruption, but also by immune remodeling within the TIME. Across different EDC classes, shared pathways such as AhR, NF-κB, ROS, PI3K–AKT–mTOR, AMPK, and PPARs may connect environmental exposure to macrophage polarization, T-cell dysfunction or exhaustion, impaired NK-cell surveillance, cytokine imbalance, and checkpoint-associated immune suppression. However, the strength of evidence varies substantially by compound class and immune endpoint. While immunotoxicity and inflammatory remodeling are supported by experimental and toxicological studies, direct evidence linking EDC exposure to cancer immunotherapy outcomes remains limited and should be regarded as an important area for future investigation.

In summary, this review bridges the critical gap between environmental toxicology and clinical oncology by delineating the multifaceted impact of EDCs on cancer progression. Beyond elucidating molecular crosstalk, we provide a translational framework that enhances clinical utility in three pivotal areas: first, the identification of EDC-associated molecular signatures offers a novel avenue for developing precision biomarkers for early cancer screening in high-exposure cohorts; second, the integration of environmental exposure history into oncological risk models enables more accurate risk stratification and the identification of patients predisposed to aggressive phenotypes; and third, the highlight of targeted interventions offers actionable strategies to mitigate EDC-induced therapeutic resistance and immune evasion. Ultimately, these insights empower clinicians to transition from conventional tumor-centric approaches to a more comprehensive, environment-aware paradigm of personalized cancer management.
